# Taxonomic note on the genus *Taiwanocantharis* Wittmer: synonym, new species and additional faunistic records from China (Coleoptera, Cantharidae)

**DOI:** 10.3897/zookeys.367.6641

**Published:** 2014-01-06

**Authors:** Yuxia Yang, Xingke Yang

**Affiliations:** 1 College of Life Sciences, Hebei University, Baoding 071002, Hebei Province, China; 2 Key Laboratory of Zoological Systematics and Evolution, Institute of Zoology, Chinese Academy of Sciences, Beijing 100101, China

**Keywords:** Cantharidae, *Taiwanocantharis*, synonym, new species, new record, China

## Abstract

*Taiwanocantharis thibetanomima* (Wittmer, 1997) is redefined and its type series is clarified. Three new speciesare described and illustrated, *Taiwanocantharis wittmeri* **sp. n.** (CHINA: Yunnan), *Taiwanocantharis adentata* **sp. n.** (CHINA: Gansu, Sichuan) and *Taiwanocantharis parasatoi* **sp. n.** (CHINA: Guangxi). *Taiwanocantharis gansosichuana* (Kazantsev, 2010) is synonymized with *Taiwanocantharis drahuska* (Švihla, 2004). *Taiwanocantharis dedicata* (Švihla, 2005) and *Taiwanocantharis malaisei* (Wittmer, 1989) are recorded to China for the first time. A key to the species of the *Taiwanocantharis thibetana* species-group is provided.

## Introduction

The genus *Taiwanocantharis* Wittmer, 1984 was upgraded from the subgenus of *Cantharis* L., 1758 by Švihla (2011). It was divided into 3 species groups, which included 16 species in total (Švihla 2011).

During our study, the type series of *Taiwanocantharis thibetanomima* (Wittmer, 1997) was shown to be plural and consist of two species, except the true *Taiwanocantharis thibetanomima* (China: Sichuan), a part of the paratypes belong to an unknown species, *Taiwanocantharis wittmeri* sp. n. (China: Yunnan). Except this, another two new species are discovered and described under the names of *Taiwanocantharis adentata* sp. n. (China: Gansu, Sichuan) and *Taiwanocantharis parasatoi* sp. n. (China: Guangxi). Now the number of the species of the *Taiwanocantharis thibetana* species-group is increased from 3 to 6, and a key is provided to distinguish them. Besides, *Taiwanocantharis gansosichuana* (Kazantsev, 2010) is considered to be a junior synonym of *Taiwanocantharis drahuska* (Švihla, 2004) based on the examination of the types of both nominal species. Additionally, *Taiwanocantharis dedicata* (Švihla, 2005) and *Taiwanocantharis malaisei* (Wittmer, 1989) are recorded to the Chinese fauna for the first time.

## Material and method

The aedeagi and abdominal sternites VIII of females were dissected under a stereoscopic microscope, cleared in 10% KOH solution for several minutes, then placed in a droplet of glycerol and examined under a compound light microscope. Photographs of the type specimens were taken with a Leica DFC320 microscope, multiple layers were stacked using CombineZM software. Line drawings were made with the aid of camera lucida attached to a Leica MZ12.5 stereomicroscope. Body length is measured from the anterior margin of the clypeus to the elytral apex, body width is measured across the humeral part of elytra.

Complete label data are listed for type specimens, using square brackets “[ ]” for our remarks and comments, [p] indicating that the following data are printed and [h] that they are handwritten. Quotation marks are used to separate data from different labels and a backslash “\” to separate data from different lines of the same label.

The material is preserved in the following collections:

CAS California Academy of Sciences, San Francisco, USA;

IZAS Institute of Zoology, Chinese Academy of Sciences, Beijing, China;

MHBU Museum of Hebei University, Baoding, China;

NHMB Naturhistorisches Museum Basel, Switzerland;

NMPC Národní muzeum, Praha, Czech Republic;

SKCR Sergey V. Kazantsev private collection, Moscow, Russia.

## Taxonomy

### A key to the species of *Taiwanocantharis thibetana* species-group

**Table:** 

1	Aedeagus: ventral process of each paramere distinctly widened, conjoint dorsal plate emarginated at lateroapical angles	*Taiwanocantharis thibetana* (Gorham, 1889)
–	Aedeagus: ventral process of each paramere narrow or slightly widened, conjoint dorsal plate not emarginated at lateroapical angles	2
2	Aedeagus: conjoint dorsal plate of parameres with the emargination of apical margin each side triangularly protuberant, the protuberance extending laterally into a short ridge on inner surface and bent ventrally	3
–	Aedeagus: conjoint dorsal plate of parameres unlike above	4
3	Pronotum with posterior angles slightly protruding, disc with a large black marking extending from anterior to posterior margin; legs uniformly black; abdominal sternite VIII of female largely emarginated in middle of posterior margin	*Taiwanocantharis satoi* (Wittmer, 1997a)
–	Pronotum with posterior angles not protruding, disc with a large central and four small prebasal black markings; legs yellow at coxae, trochanters and femora, mostly black at tibiae and tarsi; abdominal sternite VIII of female moderately emarginated in middle and slightly emarginated on both sides of posterior margin	*Taiwanocantharis parasatoi* sp. n.
4	Aedeagus: conjoint dorsal plate of parameres without any tooth on inner surface	*Taiwanocantharis adentata* sp. n.
–	Aedeagus: conjoint dorsal plate of parameres each side with a tooth near apical margin on inner surface	5
5	Each outer tarsal claw with a lobe at base in female; aedeagus: conjoint dorsal plate of parameres each side with a large tooth near apical margin on inner surface, lateroapical angles obtusely dentated	*Taiwanocantharis thibetanomima* (Wittmer, 1997b)
–	All tarsal claws simple in female; aedeagus: conjoint dorsal plate of parameres each side with a small tooth near middle of apical margin on inner surface, lateroapical angles acutely dentated	*Taiwanocantharis wittmeri* sp. n.

#### Taiwanocantharis thibetanomima

(Wittmer, 1997)

http://species-id.net/wiki/Taiwanocantharis_thibetanomima

Figs 1 , 5–8

Cantharis (s.str.) *thibetanomima* Wittmer, 1997: 294, Figs. 151–153.
Taiwanocantharis thibetanomima : Švihla 2011: 5.

##### Type material examined.

Holotype: 1 ♂ (NHMB): [h]“Sichuan, 2500m \ Emei Shan \ 29°35'N/103°11'E”, [h]“CHINA, \ 22/24.VI.1990”, [h] “Cantharis \ thibetanomima \ Wittm. \ det. W. Wittmer”, [p]“HOLOTYPUS”, [p]“CANTHARIDAE \ CANTH00004087”. Paratypes: 1 ♂ (NHMB): same data to the holotype, [p]“CANTHARIDAE \ CANTH00003067”; 1 ♀ (NHMB): same data, [p]“CANTHARIDAE \ CANTH00003209”; 1 ♀ (NHMB): same data, [p] “CANTHARIDAE \ CANTH00003565”; 1 ♂ (NHMB): [p]“CHINA / Sichuan \ 103.20el/29.30nw \ Mt. Emei 500-1200m \ 4.-18.V.1989 \ S. &J. Kolíáč leg.”, [h]“thibetanomima”, [p]“CANTHARIDAE \ CANTH00003497”.

##### Additional material examined.

CHINA: Sichuan: 1 ♂, 1 ♀ (IZAS): Emei Shan, 2100–3100m, 25.VI.1955, leg. B.R. Ou.

##### Redescription.

**Figures 1–4.**
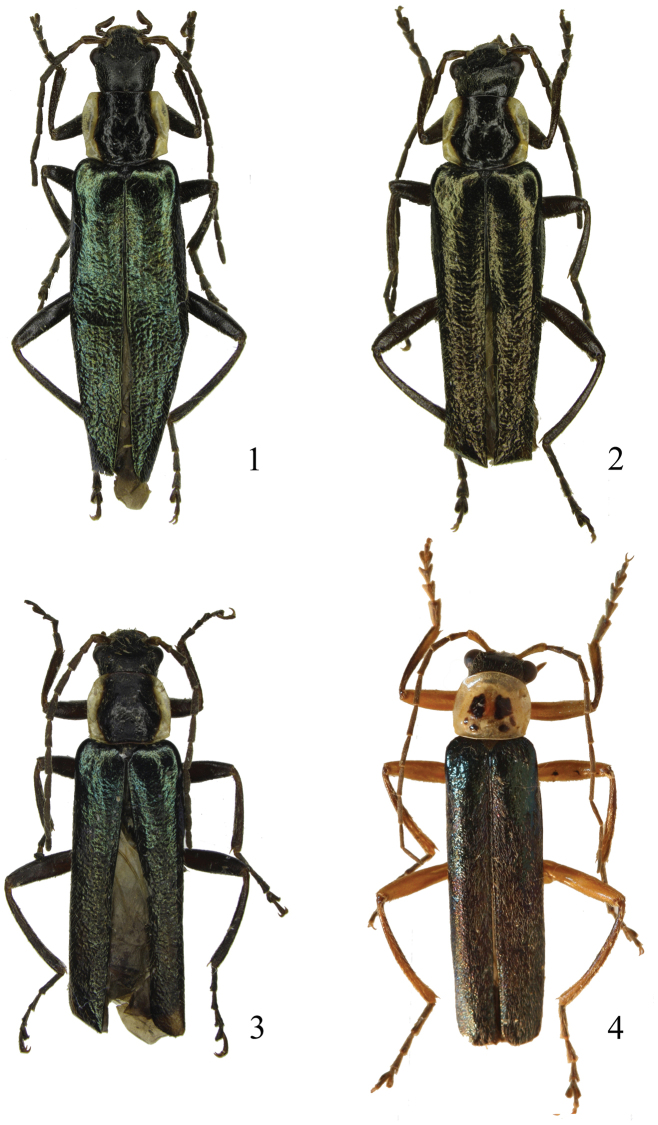
Male habitus, dorsal view: **1** *Taiwanocantharis thibetanomima* (Wittmer, 1997) **2** *Taiwanocantharis wittmeri* sp. n. **3** *Taiwanocantharis adentata* sp. n. **4** *Taiwanocantharis parasatoi* sp. n.

**Male** (Fig. 1). Body black, except mandibles dark brown, antennomeres I–II brown on ventral sides, pronotum yellow, with a large black marking extending from anterior to posterior margin, elytra green, with strongly metallic shine.

Head rounded, surface matt on frons, densely punctate on vertex, eyes slightly protruding, head width acrossing eyes distinctly narrower than pronotum; terminal maxillary palpomeres nearly long-triangular, widest at basal one-third; antennae filiform, extending to elytral middle length, antennomeres II about 3 times longer than wide, III slightly longer than II, IV–XI each with a narrow, smooth longitudinal to oval groove nearly in middle of outer margin.

Pronotum wider than long, widest at anterior one-third, anterior margin straight, lateral margins sinuate, posterior margin bisinuate and narrowly bordered, anterior angles rounded, posterior angles slightly protruding, disc slightly convex at postero-lateral parts, surface lustrous, slightly largely and sparsely punctate.

Elytra nearly parallel-sided, about 3 times longer than width at humeri, about 4 times of length of pronotum, dorsum finely punctate, lustrous at anterior one-third parts, roughly but shallowly rugoluse-lacunose on the rest.

Legs: all outer tarsal claws each with a triangular lobe at base, inner claws simple.

**Figures 5–12.**
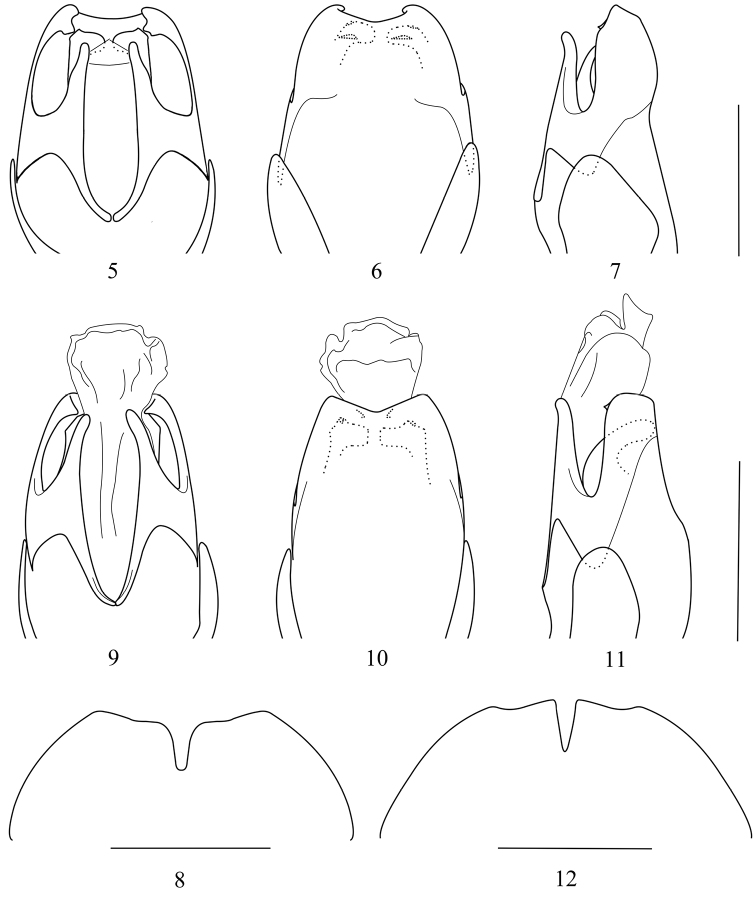
Aedeagus: (**5, 9** ventral view **6, 10** dorsal view **7, 11** lateral view) **8, 12** Abdominal sternite VIII of female, ventral view: **5–8** *Taiwanocantharis thibetanomima* (Wittmer, 1997) **9–12** *Taiwanocantharis wittmeri* sp. n. Scale bars: 1 mm.

Aedeagus (Figs 5–7): ventral process of each paramere narrow, distinctly shorter than conjoint dorsal plate; conjoint dorsal plate with apical margin widely emarginated in middle, lateroapical angles obtusely dentated, each side with a large tooth near apical margin on inner surface; laterophyse adhered to median lobe, with apex bent towards middle, the portion around the bending corner with upper margin slightly protuberant and bent dorsally.

**Female.** Similar to male, but eyes smaller, terminal maxillary palpomeres shorter, nearly widest in middle, antennae shorter, extending to elytral one-third length, antennomeres IV–XI without any groove, pronotum wider, elytra with lateral margins slightly diverging posteriorly, abdominal sternite VIII (Fig. 8) largely emarginated in middle and slightly emarginated on both sides of posterior margin, the portion between middle and each lateral emarginations rounded at apex.

Body length: 9.0–11.0 mm; width: 2.0–2.5 mm.

##### Distribution.

China (Sichuan). Excluded from Yunnan province at the moment.

##### Remarks.

In the study, the type series of this species were discovered to be plural and consist of two species. Except the true *Taiwanocantharis thibetanomima*, which was located in Sichuan, China, the paratypes from Yunnan, China designated in the original publication belong to another unknown species described below, *Taiwanocantharis wittmeri* sp. n. Besides, the photos of aedeagus provided by Wittmer (1997b) is not of *Taiwanocantharis thibetanomima* but *Taiwanocantharis wittmeri* sp. n. So it is necessary to redescribe and illustrate this species here.

#### Taiwanocantharis wittmeri sp. n.

http://zoobank.org/718F1778-CB50-4A92-AA39-74D0695B6AF7

http://species-id.net/wiki/Taiwanocantharis_wittmeri

Figs 2 , 9–12

Cantharis (s.str.) *thibetanomima* Wittmer, 1997b: 294, Figs. 151–153, parte.

##### Type material.

Holotype♂ (NHMB): [p] “CHINA, Yunnan prov. \ 27°08'N 100°14'E, 2900 \ Yulongshan mts., -3500m \ Baishui 7.-12.VII.1990 \ Vít Kubáň leg.”, [p] “CANTHARIDAE \ CANTH00003678”. Paratypes: 1 ♀ (NHMB): same data to holotype, [p] “CANTHARIDAE \ CANTH00003695”; 1 ♀ (NHMB): same data, [p] “CANTHARIDAE \ CANTH00003057”; 1 ♀ (NHMB): same data, [p] “CANTHARIDAE \ CANTH00002059”; 1♂ (NHMB): [p] “YUNNAN, 23-24.JUN. \ Yulong Mts., 1993 \ 27.00N 100.12E \ Bolm lgt. 3200m”, [p] “CANTHARIDAE \ CANTH00003523”; 1 ♀ (NHMB): [p] “YUNNAN, 24-26 May \ Yulong mts., 1993 \ 27.01N 100.12E \ Bolm lgt. 3200m”, [p] “CANTHARIDAE \ CANTH00003665”; 1 ♀ (IZAS): [p] “YUNNAN 3300-3500m \ 27.07N 100.13E 1993\ YULONGSHAN mts. 20- \ Vít Kubáň leg. -21/6.”, [p] “IOZ(E) 217863”; 1 ♂ (IZAS): [p] “China N-YUNNAN \ 27°08'N 100°14'E \ Yulongshan mts. 2900- \ -3500m BAISHUI vill. \ lgt. D. Král 7-12/7’90”, [p] “IOZ(E) 217864”; 1 ♀ (NHMB): [p] “China Yunnan 1-19.VII \ HEISHUI 27.13N 100.19E \ 35km N of Lijiang \ legit S. Beĉváŕ 1992”, [p] “CANTHARIDAE \ CANTH00003632”; 1 ♂ (NHMB): [p] “CHINA: N. Yunnan \ 30km N of Lijiang \ 3000m, 3.VII.1990 \ L.& M. Bocák. lgt.”, [p] “CANTHARIDAE \ CANTH00004071”; 1 ♀ (NHMB): [p] “YUNNAN 2500-3100m \ 25.58N 100.21E 30/5 \ Jizushan mts. -3/6 \ Vit Kubáň leg. 1993”, [p] “CANTHARIDAE \ CANTH00003636”; 1 ♀ (NHMB): same data, [p] “CANTHARIDAE \ CANTH00003773”; 1 ♂ (NHMB): [p] “YUNNAN, 30 May-3 Jun \ JIZU MTS., 1993 \ 25.58N 100.21E \ Bolm lgt. 2800m”, [p] “CANTHARIDAE \ CANTH00003608”; 1 ♀ (NHMB): same data, [p] “CANTHARIDAE \ CANTH00003804”; 1 ♀ (NHMB): [p] “YUNNAN 2000-2800m \ 25.11N 100.24E \ Weibaoshan mts. \ W slope 25-28/6.92 \ David Král leg.”, [p] “CANTHARIDAE \ CANTH00003651”.

##### Description.

**Male** (Fig. 2). Body black, except mandibles dark brown, antennomeres I–II brown on ventral sides, pronotum yellow, with a large black marking extending from anterior to posterior margin, elytra mixed green and bronze, with strongly metallic shine.

Head rounded, surface matt on frons, densely punctate on vertex, eyes slightly protruding, head width acrossing eyes distinctly narrower than pronotum; terminal maxillary palpomeres nearly long-triangular, widest at basal one-third; antennae filiform, extending to elytral middle length, antennomeres II about 3 times longer than wide, III slightly longer than II, IV–XI each with a narrow, smooth longitudinal to oval groove nearly in middle of outer margin.

Pronotum wider than long, widest at anterior one-third, anterior margin straight, lateral margins sinuate, posterior margin bisinuate and narrowly bordered, anterior angles rounded, posterior angles nearly rectangular, not protruding, disc slightly convex at postero-lateral parts, surface lustrous, finely and sparsely punctate.

Elytra nearly parallel-sided, about 3 times longer than width at humeri, about 3.5 times of length of pronotum, dorsum finely punctate, lustrous at anterior one-third parts, roughly but shallowly rugoluse-lacunose on the rest.

Legs: all outer tarsal claws each with a triangular lobe at base, inner claws simple.

Aedeagus (Fig. 9–11): ventral process of each paramere narrow, nearly as long as conjoint dorsal plate; conjoint dorsal plate with apical margin triangularly emarginated in middle, lateroapical angles acutely dentated, each side with a small tooth near middle of apical margin on inner surface; laterophyse adhered to median lobe, with apex bent towards middle, the portion around the bending corner with upper margin slightly protuberant and bent dorsally.

**Female.** Similar to male, but eyes smaller, terminal maxillary palpomeres shorter, nearly widest in middle, antennae shorter, extending to elytral one-third length, antennomeres IV–XI without any groove, pronotum wider, elytra with lateral margins slightly diverging posteriorly, all tarsal claws simple, abdominal sternite VIII (Fig. 12) largely emarginated in middle and slightly emarginated on both sides of posterior margin, the portion between middle and each lateral emarginations tapered at apex.

Body length: 8.0–11.0 mm; width: 2.0–2.5 mm.

##### Diagnosis.

This species is similar to *Taiwanocantharis thibetanomima*, but can be distinguished from the latter by the following characters: all tarsal claws simple in female; aedeagus: ventral process of each paramere nearly as long as conjoint dorsal plate, conjoint dorsal plate with lateroapical angels acutely dentated, each side with a small tooth near middle of apical margin on inner surface; abdominal sternite VIII of female with the portions between middle and lateral emarginations tapered at apex.

##### Distribution.

China (Yunnan).

##### Etymology.

This species is named after the late distinguished taxonomist Dr. Walter Wittmer.

##### Remarks.

This new species was described based on a part of the paratypes, which are located in Yunnan, China, of *Taiwanocantharis thibetanomima*. What’s noted, two paratypes provided with the information as “Yulongshan Mts., Baishui, 2900–3500m, 27°08'N, 100°14'E, 7.–12.VII.1990, 22.–24.VI.1993”, according to the original publication by Wittmer (1997), can not found in the collection of NHMB. But four not three paratypes labeled as “Jizushan Mts., 2500–3100m, 25°58'N, 100°21'E, 30.V.–3.VI.1993” were found in NHMB, all of them were designated as the paratypes of *Taiwanocantharis wittmeri* sp. n.

#### Taiwanocantharis adentata sp. n.

http://zoobank.org/F6B46DF0-FD93-4F9A-ABDC-7018B5C87221

http://species-id.net/wiki/Taiwanocantharis_adentata

Figs 3 , 13–16

##### Type material.

Holotype♂ (MHBU): CHINA: Gansu: Wenxian, Huangtuling, 2350m, 9.VII.2003, leg. Y.B. Ba & Y. Yu. Paratypes: 1 ♀ (MHBU): same data to holotype; 1 ♀ (MHBU): same data, 8.VII.2003. 1 ♂ (NHMB): [Sichuan]: Da Tsien Lou [Kangding], 3.VI.(18)93 (collector, hand-writing, hardly readable).

##### Description.

**Male** (Fig. 3). Body black, except mandibles brown, antennomeres I–II brown on ventral sides, pronotum yellow, with a large black marking extending from anterior to posterior margin, elytra green, with strongly metallic shine.

Head rounded, surface matt on frons, densely punctate on vertex, eyes slightly protruding, head width acrossing eyes slightly narrower than anterior margin of pronotum; terminal maxillary palpomeres nearly long-triangular, widest at basal one-third; antennae filiform, extending to elytral middle length, antennomeres II about 3 times longer than wide, III slightly longer than II, IV–XI each with a narrow, smooth longitudinal to oval groove nearly in middle of outer margin.

Pronotum wider than long, widest at anterior one-third, anterior margin straight, lateral margins sinuate, posterior margin bisinuate and narrowly bordered, anterior angles rounded, posterior angles nearly rectangular, not protruding, disc slightly convex at postero-lateral parts, surface lustrous, finely and sparsely punctate.

Elytra nearly parallel-sided, about 3 times longer than width at humeri, about 4 times of length of pronotum, dorsum finely punctate, lustrous at anterior one-third parts, roughly but shallowly rugoluse-lacunose on the rest.

Legs: all outer tarsal claws each with a triangular lobe at base, inner claws simple.

**Figures 13–20.**
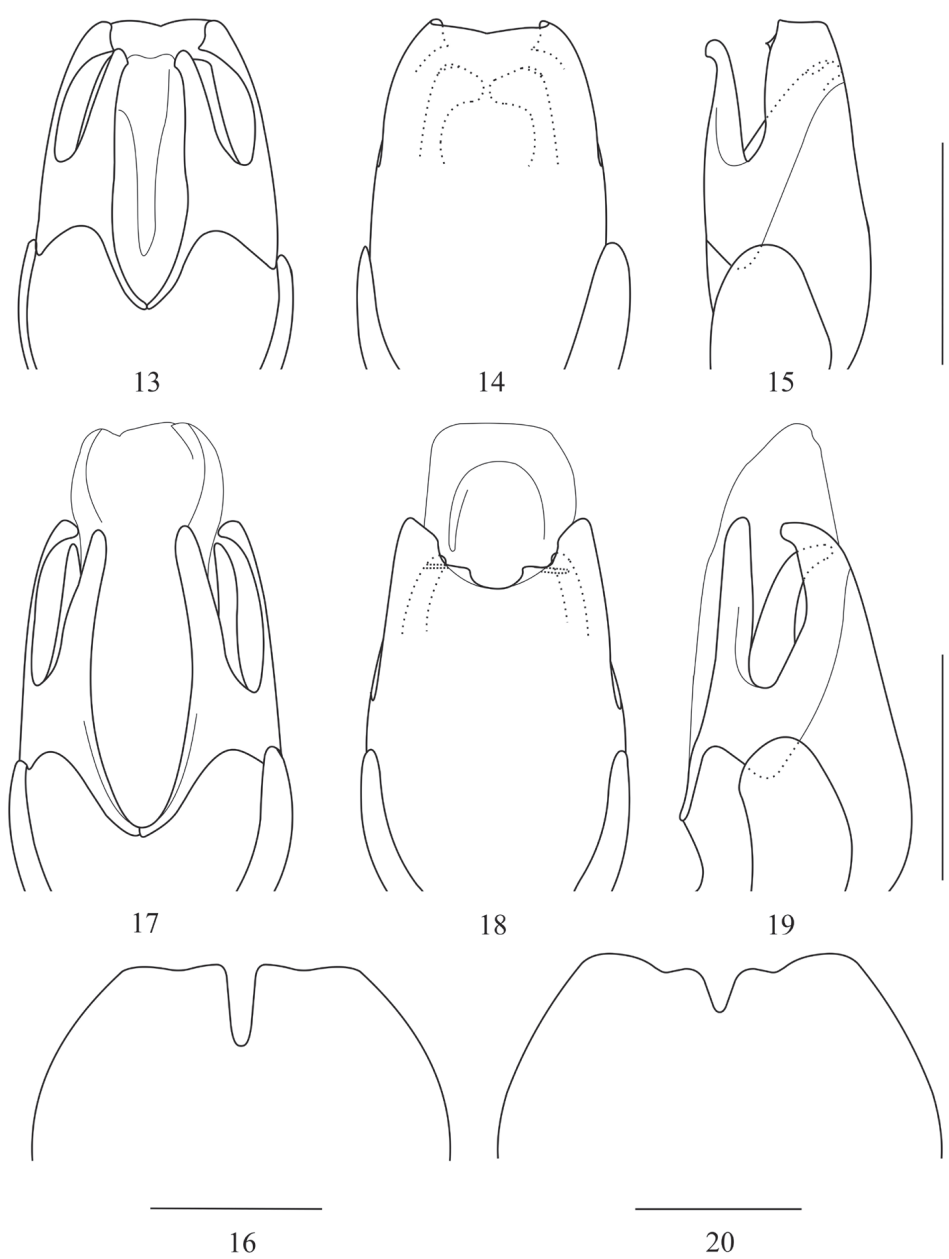
Aedeagus: (**13, 17** ventral view **14, 18** dorsal view **15, 19** lateral view) **16, 20** Abdominal sternite VIII of female, ventral view: **13–16** *Taiwanocantharis adentata* sp. n. **17–20** *Taiwanocantharis parasatoi* sp. n. Scale bars: 1 mm.

Aedeagus (Figs 13–15): ventral process of each paramere narrow, slightly shorter than conjoint dorsal plate; conjoint dorsal plate with apical margin slightly emarginated in middle, lateroapical angles acutely dentated, without any tooth on inner surface; laterophyse adhered to median lobe, with apex bent towards middle, the portion around the bending corner with upper margin slightly protuberant and bent dorsally.

**Female.** Similar to male, but eyes smaller, terminal maxillary palpomeres shorter, nearly widest in middle, antennae shorter, extending to elytral one-third length, antennomeres IV–XI without any groove, pronotum wider, elytra with lateral margins slightly diverging posteriorly, abdominal sternite VIII (Fig. 16) largely emarginated in middle and slightly emarginated on both sides of posterior margin, the portion between middle and each lateral emarginations subrounded at apex.

Body length: 8.0–11.0 mm; width: 2.0–2.5 mm.

##### Diagnosis.

This species is similar to *Taiwanocantharis thibetanomima*, but can be distinguished from the latter by the aedeagus: conjoint dorsal plate of parameres with lateroapical angles acutely dentated, without any tooth on inner surface.

##### Distribution.

China (Gansu, Sichuan).

##### Etymology.

This specific name is derived from the Latin “*a*-” (none) + “*dentatus*” (toothed), referring to its conjoint dorsal plate of parameres without any tooth on inner surface.

#### Taiwanocantharis parasatoi sp. n.

http://zoobank.org/CD261DB4-3587-40AB-8343-CB2FF845B8D0

http://species-id.net/wiki/Taiwanocantharis_parasatoi

Figs 4 , 17–20

##### Type material.

Holotype♂ (MHBU): CHINA: Guangxi: Wuming, Damingshan, 1100m, 27.V.2011, leg. H.Y. Liu. Paratypes: 2 ♂♂, 1 ♀(MHBU): same data to holotype; 2♂♂(MHBU): same locality, 600–900 m, 25.V.2011, leg. H.Y. Liu; 1 ♂, 1 ♀(MHBU): same locality, 1230–1423m, 20.V.2011, leg. H.Y. Liu.

##### Description.

**Male** (Fig. 4). Head black, mouthparts yellow, slightly darkened at terminal maxillary and labial palpomeres, mandibles dark brown, antennae black, antennomeres I–II yellow on ventral sides, pronotum yellow, disc with two central and four prebasal small black markings, scutellum yellow, elytra green, with strongly metallic shine, thorax and abdomen yellow on ventral sides, abdominal sternites II–VIII each side with a small round black marking, legs yellow, tibiae black, with apical parts yellow on ventral sides, tarsi black.

Head rounded, surface lustrous, densely punctate on vertex, eyes strongly protruding, width acrossing eyes slightly narrower than pronotum; terminal maxillary palpomeres nearly long-triangular, widest at basal one-third; antennae filiform, extending to elytral middle length, antennomeres II about 3 times longer than wide, III slightly longer than II, IV–XI each with a narrow, smooth longitudinal to oval groove nearly in middle of outer margin.

Pronotum distinctly wider than long, widest at anterior one-third, anterior margin arcuate, lateral margins slightly sinuate, posterior margin arcuate and narrowly bordered, anterior angles rounded, posterior angles subrounded, not protruding, disc slightly convex at postero-lateral parts, surface lustrous, finely and sparsely punctate.

Elytra nearly parallel-sided, about 4 times longer than width at humeri, about 5 times of length of pronotum, dorsum finely punctate, lustrous at humeral parts, roughly but shallowly rugoluse-lacunose on the rest.

Legs: slender, all outer tarsal claws each with a triangular lobe at base, inner claws simple.

Aedeagus (Figs 17–19): ventral process of each paramere slightly widened, nearly as long as conjoint dorsal plate; conjoint dorsal plate slightly bent ventrally at apex in lateral view, with apical margin roundly emarginated in middle, each side of the emargination triangularly protuberant, the protuberance extending laterally into a short ridge on inner surface and bent ventrally, lateroapical angles blunt-coniformly dentated; laterophyses normal and separated on both sides of median lobe, with apices rounded.

**Female.** Similar to male, but eyes smaller, terminal maxillary palpomeres shorter, nearly widest in middle, antennae shorter, extending to elytral one-third length, antennomeres IV–XI without any groove, elytra with lateral margins slightly diverging posteriorly, abdominal sternites II–VII each side with a small round black marking, VIII (Fig. 20) moderately emarginated in middle and slightly emarginated on both sides of posterior margin, the portion between middle and each lateral emarginations rounded at apex.

Body length: 12.0–14.0 mm; width: 2.0–3.0 mm.

##### Diagnosis.

This new species is related to *Taiwanocantharis satoi* (Wittmer, 1997a), but can be distinguished from the latter by the following characters: pronotum with posterior angles not protruding, disc with a large central and four small prebasal black markings; legs yellow at coxae, trochanters and femora, black at tibiae and tarsi; abdominal sternite VIII of female moderately emarginated in middle and slightly emarginated on both sides of posterior margin.

##### Distribution.

China (Guangxi).

##### Etymology.

This specific name is derived from the Greek prefix “*para*-” (similar), referring to its close relationship to *Taiwanocantharis satoi*.

#### Taiwanocantharis drahuska

(Švihla, 2004)

http://species-id.net/wiki/Taiwanocantharis_drahuska

Cordicantharis drahuska Švihla, 2004: 176, Figs 55–57, 198.
Cantharis (s.str.) *gansosichuana* Kazantsev, 2010: 154, Figs 2–4.
Taiwanocantharis drahuska : Švihla 2011: 5.
Taiwanocantharis gansosichuana : Švihla 2011: 5. **syn. n.**

##### Type material examined.

*Cordicantharis drahuska*: Holotype: 1 ♂ (NMPC): [p]“China: Shaanxi, 2.-4.7.1998 \ Qing Ling Shan mts., 3500m \ Hou Zen Zi-Tai Bai \ Jindra, Trýzna&Šafránek lgt.”, [p]“HOLOTYPUS \ Cordicantharis \ drahuska sp. n. \ V. Švihla det. 2003”.

*Cantharis* (s.str.) *gansosichuana*: Holotype: 1 ♂ (SKCR): [p]“CHINA: S Gansu \ Tepo [Tewo] 2500-2800m \ 26-28/vi/2001 \ S. Kazantsev leg.”, [p-h]“Cantharis \ gansosichuana \ sp. n. \ S. Kazantsev det. 2010”, [p]“HOLOTYPUS \ S. Kazantsev des.”.

##### Additional material examined.

CHINA: Sichuan: 2 ♂♂, 1 ♀ (NMPC): Erlangshan Mts., 2600m, E Luding, 14.–16.VI.2003, leg. S. Murzin. Gansu: 1 ♂, 6 ♀♀ (HBUM): Wenxian, Huangtuling, 2350m, 9.VII.2003, leg. Yi-Bin Ba & Yang Yu; 1 ♀ (HBUM): same data, 8.VII.2003. Ningxia: 1 ♂ (HBUM): Heshangpu Forestry center, 1.–2.VII.2008, leg. X.P. Wang & X.L. Liu; 1 ♀ (HBUM): same data, 2100m, 5.–6.VII.2008; 2 ♂♂, 13 ♀♀ (HBUM): Jingyuan, Erlonghe, 3.–4.VII.2009, leg. X.P. Wang & X.L. Zhao; 4 ♀♀ (HBUM): same locality, 23.VI.2008, leg. Hong-Fan Ran; 2 ♀♀ (HBUM): same locality, 6.VII.2009, leg. S.Y. Zhou & X.J. Meng; 1 ♀ (HBUM): same locality, 6.VII.2009, leg. H.F. Ran & S.S. Zhang; 4 ♀♀ (HBUM): same locality, 23.VI.2008, leg. H.F. Ran; 1 ♂, 4 ♀♀ (HBUM): Jingyuan, Xixia Forestry center, 9.–10.VII.2009, leg. X.P. Wang & X.L. Zhao; 1 ♂, 3 ♀♀ (HBUM): same locality, 27.–28.VII.2008, leg. X.M. Li, H.F. Ran & Q.Q. Wu; 3 ♂♂, 8 ♀♀ (HBUM): same data, 15.–16.VII.2008; 1 ♂ (HBUM): same locality, 27.VII.2008, leg. F. Yuan; 2 ♀♀ (HBUM): Wanghuanan Forestry center, 20.VI.2008, leg. H.F. Ran; 1 ♀ (HBUM): same locality, 3.–4.VII.2009, leg. G.D. Ren & Y.W. Ba; 2 ♀♀ (HBUM): Jingyuan, Dongshanpo, 8.VII.2009, leg. H.F. Ran & S.S. Zhang; 1 ♀ (HBUM): Hongxia Forestry center, 25.VI.2008, leg. H.F. Ran; 1 ♂ (HBUM): Longde, Fengtai Forestry center, 29.–30.VI.2008, leg. X.M. Li, H.F. Ran & Q.Q. Wu.

##### Distribution.

China (Sichuan, Gansu, Shaanxi, Ningxia). Newly recorded for Ningxia Hui Autonomous Region.

##### Remarks.

It had been noted by Švihla (2011) that *Taiwanocantharis gansosichuana* (Kazantsev, 2010) was possibly a junior synonym of *Taiwanocantharis drahuska* (Švihla, 2004) without examining the types of the former species. During our study, the holotypes of the both nominal species and a large series of additional material at our disposal were examined, and no difference between them was discovered, thereby we suggest *Taiwanocantharis drahuska* be synonymized with *Taiwanocantharis gansosichuana*.

#### Taiwanocantharis dedicata

(Švihla, 2005)

http://species-id.net/wiki/Taiwanocantharis_dedicata

Cantharis (s.str.) *dedicata* Švihla, 2005: 88, Figs 35–37.
Taiwanocantharis dedicata : Švihla 2011: 5.

##### Type material examined.

Holotype: 1 ♂ (NMPC): [p]“Laos: Hua Phan prov. \ Ban Saluei, Mt. Phu Phan \ 20.13°N, 103.95°E, 1300-2000m \ F. & L. Kantner lgt. \ 6-17.v.2004”, [p]“HOLOTYPUS \ Cantharis (s.str.) \ dedicata sp. n. \ V. Švihla det. 2005”.

##### Additional material examined.

CHINA: Yunnan: 2 ♂♂ (IZAS):Tengchong, Jietou town, Shaba, 1850m, 25.3926°N, 98.7035°E, 25.V.2006, leg. H.B. Liang & P. Hu.

##### Distribution.

China (new record: Yunnan); Laos.

#### Taiwanocantharis malaisei

(Wittmer, 1989)

http://species-id.net/wiki/Taiwanocantharis_malaisei

Cantharis malaisei Wittmer, 1989: 212, Figs. 5, 7.
Taiwanocantharis malaisei : Švihla 2011: 5.

##### Type material examined.

Holotype: 1 ♂ (NHMB): [p]“N.E. BURMA **\** Kambaiti, 7000ft \ 3/5 1934 \ R. Malaise”, [h]“Canthris \ malaisei \ Wittm. \ det. W. Wittmer”,[h]“HOLOTYPUS”, [p]“Naturhistorisches \ Museum Basel \ Coll. W. Wittmer”, [p]“CANTHARIDAE \ CANTH00003352”. Paratypes: 2 ♀♀ (NHMB): same data, 3. –7.V.1934; 1 ♀ (NHMB): same data, 12.IV.1934; 2 ♀♀ (NHMB): same data, 4.–8.VI.1934; 1 ♂, 1 ♀ (NHMB): same data, 2000m, 27.V.1934.

##### Additional material examined.

CHINA: Yunnan: 1 ♀ (NHMB): Gaoligong mts., 2200–2500m, 24°57'N, 98°45'E, 8.–16.V.1995, leg. Vit Kubáň; 1 ♀ (CAS): Lushui County, Pianma Township, Gangfang, 1675m, 26.12070°N/098.57830°E, 16.V.2005, attracted to uv and mv lights at night, Stop# HBL-05-11, H. B. Liang collector [CASENT 1036703].

##### Distribution.

China (new record: Yunnan); Myanmar.

## Supplementary Material

XML Treatment for Taiwanocantharis thibetanomima

XML Treatment for Taiwanocantharis wittmeri

XML Treatment for Taiwanocantharis adentata

XML Treatment for Taiwanocantharis parasatoi

XML Treatment for Taiwanocantharis drahuska

XML Treatment for Taiwanocantharis dedicata

XML Treatment for Taiwanocantharis malaisei
